# Surface passivated halide perovskite single-crystal for efficient photoelectrochemical synthesis of dimethoxydihydrofuran

**DOI:** 10.1038/s41467-021-21487-8

**Published:** 2021-02-22

**Authors:** Xu-Dong Wang, Yu-Hua Huang, Jin-Feng Liao, Ze-Feng Wei, Wen-Guang Li, Yang-Fan Xu, Hong-Yan Chen, Dai-Bin Kuang

**Affiliations:** MOE Key Laboratory of Bioinorganic and Synthetic Chemistry, Lehn Institute of Functional Materials, School of Chemistry, Sun Yat-sen University, Guangzhou, P. R. China

**Keywords:** Artificial photosynthesis, Photocatalysis, Nanoscale materials

## Abstract

Halide perovskite single-crystals have recently been widely highlighted to possess high light harvesting capability and superior charge transport behaviour, which further enable their attractive performance in photovoltaics. However, their application in photoelectrochemical cells has not yet been reported. Here, a methylammonium lead bromide MAPbBr_3_ single-crystal thin film is reported as a photoanode with potential application in photoelectrochemical organic synthesis, 2,5-dimethoxy-2,5-dihydrofuran. Depositing an ultrathin Al_2_O_3_ layer is found to effectively passivate perovskite surface defects. Thus, the nearly 5-fold increase in photoelectrochemical performance with the saturated current being increased from 1.2 to 5.5 mA cm^−2^ is mainly attributed to suppressed trap-assisted recombination for MAPbBr_3_ single-crystal thin film/Al_2_O_3_. In addition, Ti^3+^-species-rich titanium deposition has been introduced not only as a protective film but also as a catalytic layer to further advance performance and stability. As an encouraging result, the photoelectrochemical performance and stability of MAPbBr_3_ single-crystal thin film/Al_2_O_3_/Ti-based photoanode have been significantly improved for 6 h continuous dimethoxydihydrofuran evolution test with a high Faraday efficiency of 93%.

## Abstract (web summary)

Perovskite single-crystal thin films inherit the advantages of low trap-states, well-defined thickness and remarkable stability. Now, researchers successfully employed MAPbBr_3_ single-crystal thin film as photoanode in the photoelectrochemical production of organic 2,5-dimethoxy-2,5-dihydrofuran.

## Introduction

In the past decade, the vast latent capacity of halide perovskites in solar energy utilization has been reported; e.g., perovskite solar cells showed a power conversion efficiency of 25.5%^1^. In addition to solar-to-electricity conversion, the high light harvesting capability, superior carrier transport, and suitable band position of halide perovskites motivate the wide applications of these materials in photocatalysis and photoelectrochemical (PEC) fields^2–6^. Compared to particulate photocatalytic systems, PEC systems feature semiconductor photocatalysts and external biases and thereby exhibit significant advantages in high-efficiency charge separation and collection as well as easy catalyst recycling^7^. Recently, halide perovskite polycrystalline thin films (PCTFs) have been exploited for PEC water splitting and CO_2_ reduction^8–17^. The use of organic solvent instead of water not only is expected to improve the stability of photoelectrode^18^, but also enables a broader set of organic synthesis reactions, achieving higher Faradaic efficiency and value-added chemicals^19–25^. Compared to PCTFs, perovskite single-crystal thin films (SCTFs) inherit the advantages of both polycrystalline films and bulk single crystals^26^, such as low trap-state densities, long carrier diffusion lengths, well-defined thicknesses and, more importantly, high stability, which thus has pinned great expectation as more promising photoelectrodes for PEC applications.

2,5-Dimethoxy-2,5-dihydrofuran (DMDF) has been considered a key intermediate for producing pyridines, pyridazines, pyrroles, benzenoid compounds, and coumarin analogs^27–30^. In its traditional chemical synthetic method, a large amount of bromine is employed as an oxidant, the mass usage of which raises issues of environmental pollution^29^. As an alternative approach to produce DMDF, electrochemical oxidation of furan can be mildly implemented in NH_4_Br alcoholic solution. In 2017, Sayama and co-worker provided another more direct approach based on a PEC system using BiVO_4_/WO_3_ as a photoanode with a Br^+^/Br^−^ mediator under visible light irradiation^30^. PEC synthesis has been found to significantly lower the required applied potential; however, the resulting photocurrent density is slightly unsatisfactory (<0.55 mA cm^−2^). Currently, it is reasonable to expect that halide perovskite SCTFs could deliver advanced PEC performance towards DMDF production, considering their remarkable photoelectric properties. However, the utilization of perovskite-based PEC cells for organic synthesis remains unexplored.

Herein, methylammonium lead bromide MAPbBr_3_ SCTF grown on a conductive FTO glass substrate is demonstrated as an efficient photoanode in a PEC cell to afford a high turnover rate for artificial photosynthesis of DMDF from furan. The surface is the most vulnerable to defects and passivation of single-crystal surface defects is the most important task. The subsequent deposition of Al_2_O_3_ and Ti overlayers significantly promoted the operating stability and photocatalytic activity of MAPbBr_3_ SCTFs. As an encouraging result, the highest photocurrent of 7.8 mA cm^−2^ (0.8 V vs. Ag/AgCl) is achieved for MAPbBr_3_ SCTF/Al_2_O_3_/Ti. Such work paves the way for the future design of high-performance perovskite SCTF-based solar-driven photosynthesis systems.

## Results and discussion

### PEC characterizations of MAPbBr_3_ SCTF and PCTF

**Fig. 1:**
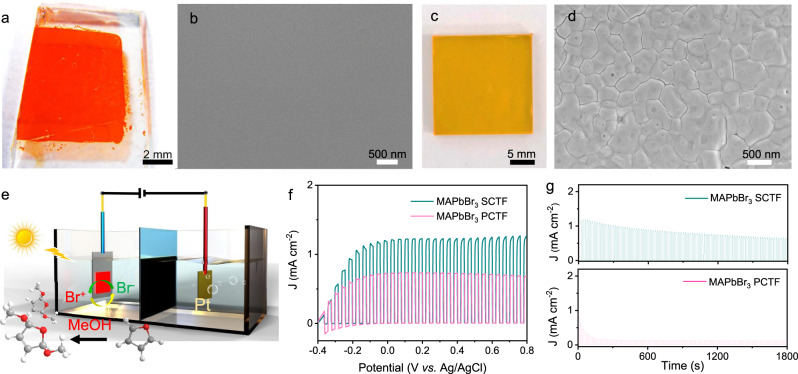
Comparison of MAPbBr_3_ SCTF with PCTF. Optical microscopy and SEM images of MAPbBr_3_ SCTF (**a**, **b**) and PCTF (**c**, **d**). **e** Schematic configuration of the proposed working principle of MAPbBr_3_ SCTF-based (or PCTF-based) PEC cells. **f** Linear sweep voltammetry (LSV) of MAPbBr_3_ SCTF-based and PCTF-based photoelectrodes. **g** Chronoamperometric trace of MAPbBr_3_ SCTF-based and PCTF-based photoelectrodes recorded at an applied potential of 0.2 V vs. Ag/AgCl.

MAPbBr_3_ SCTFs were grown in situ on an FTO/TiO_2_ substrate through our previously reported space-limited crystallization method with some modifications^31,32^. The as-prepared MAPbBr_3_ SCTF exhibits a smooth and pinhole-free surface with a lateral size of 11.5 mm × 7.6 mm and a film thickness of approximately 14 µm (Fig. 1a, b and Supplementary Fig. 1a). In contrast, a 300-nm-thick MAPbBr_3_ PCTF prepared by spin-coating (Fig. 1c and Supplementary Fig. 1b) is composed of small crystals accompanied by abundant sharp grain boundaries (Fig. 1d). The configuration of the PEC cell system is depicted in Fig. 1e, where MAPbBr_3_ SCTF (or PCTF) on an FTO/TiO_2_ substrate and a Br^−^/Br^+^ redox couple in an acetonitrile/methanol mixed solution function as the photoanode and electrolyte, respectively. Ultraviolet photoelectron spectra (UPS, Supplementary Fig. 2a, b) show that the valence band maximum (VBM) of the MAPbBr_3_ SCTF is approximately 1.45 V vs. SHE, capable of bromine oxidation (0.7 V vs. SHE, Supplementary Fig. 2c)^28^. Upon light excitation, the Br^−^ in the electrolyte will be oxidized into Br^+^ species by photogenerated holes, which then serve as oxidizing reagents to transform furan into DMDF. The resultant saturated photocurrent densities of MAPbBr_3_ PCTF and SCTF are 0.7 and 1.2 mA cm^−2^ at 0.20 V vs. Ag/AgCl (Fig. 1f), respectively. Such a 70% enhancement in photocurrent density for MAPbBr_3_ SCTF is mainly ascribed to its high crystallinity, high charge mobility and low trap density^31^. In addition, it can be clearly seen that the photocurrent density of the MAPbBr_3_ PCTF PEC cell decreases rapidly within ~3 min, while that of the MAPbBr_3_ SCTF retains 54% of the initial value even after 30 min (Fig. 1g). This phenomenon is further supported by the stability of the materials in acetonitrile/methanol electrolyte (Supplementary Fig. 3). MAPbBr_3_ PCTF decomposed within 25 min, while MAPbBr_3_ SCTFs exhibit negligible degradation even after 20 h, further demonstrating the better stability of SCTFs. The diffusion length of MAPbBr_3_ SCTF (5.6 µm)^31^ is much higher than that of perovskite-based thin films (0.33 µm)^33^. Electric fields can help carriers migrate from a 14 µm thick single crystal to the surface and then participate in chemical reactions. On the premise of ensuring light absorption, further reducing the thickness of single crystal film is beneficial for shortening the carrier transmission distance and further improving the PEC performance. However, it is still challenging to control the perovskite single crystal films with large size and several microns thickness. In terms of influence of thickness on stability of perovskite film, thicker films tend to exhibit better stability (Supplementary Fig. 4). However, grain boundaries in PCTF have also been widely proven to primarily bear the brunt of perovskite degradation due to massively distributed trap states^34^. Therefore, the stability of the SCTF-based PEC cell can be mainly ascribed to the advantages of being few defect states.

### Investigation of the Al_2_O_3_ passivation effect for MAPbBr_3_ SCTFs

Halide perovskite materials have been widely proven to have a low trap formation energy and reconfigurable surface lattice^35,36^, and therefore, surface trap states are easily formed as charge carrier recombination centers^37^. Herein, we deposit an ultrathin Al_2_O_3_ layer onto a MAPbBr_3_ SCTF through atomic layer deposition (ALD) to further boost the PEC performance by reducing the amounts of surface trap states (see section “Experimental details” in the “Methods” section).

**Fig. 2:**
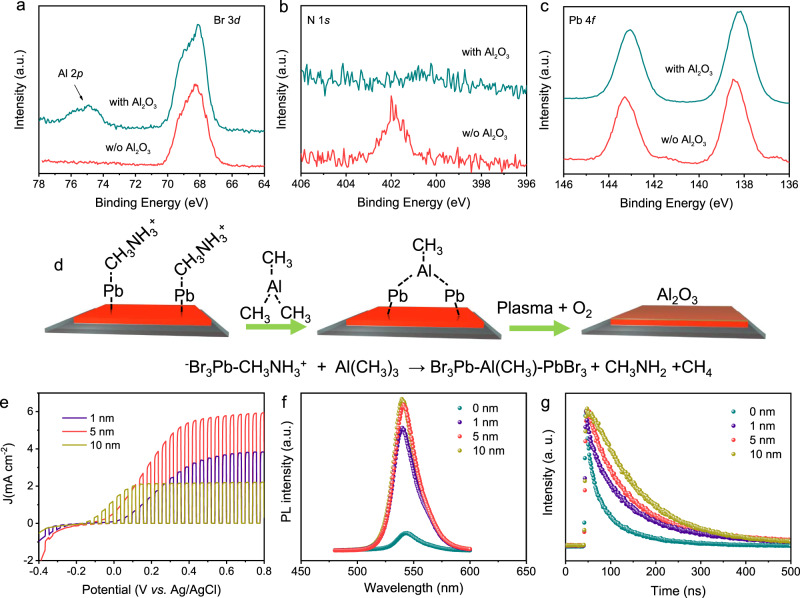
Al_2_O_3_ passivation effect investigation. XPS spectra of pristine MAPbBr_3_ SCTF and MAPbBr_3_ SCTF/Al_2_O_3_: Al 2*p* and Br 3*d* (**a**), N 1*s* (**b**), and Pb 4*f* (**c**). **d** Proposed passivation mechanism of Al_2_O_3_ on the MAPbBr_3_ SCTF surface. **e** PEC behavior of MAPbBr_3_ SCTF/Al_2_O_3_-based photoelectrodes as a function of Al_2_O_3_ deposition thickness. Al_2_O_3_-thickness-dependent steady-state photoluminescence (PL) spectra (**f**) and time-resolved PL decay curves (**g**).

The presence of Al_2_O_3_ is first verified by the additional Al 2*p* peak at 74.8 eV in the X-ray photoelectron spectroscopy (XPS) spectra (Fig. 2a). Furthermore, characteristic signals of Al–CH_3_, (OH)–Al=O and Al–O–Al species are observed at 1256, 1080, and 888 cm^−1^ in the Fourier transform infrared (FTIR) spectra after ALD treatment, again proving the existence of Al_2_O_3_ (Supplementary Fig. 5a). During the process of ALD deposition of Al_2_O_3_, TMA reacts with CH_3_NH_3_^+^ of MAPbBr_3_ and results in CH_3_NH_2_ release accompanied by the formation of Br_3_Pb–Al(CH_3_)–PbBr_3_ intermediates (Fig. 2d)^38^, which further react with O_2_ to produce Al_2_O_3_. Further insight into the chemical modification on the perovskite surface was provided by comparing the surface XPS spectra of N 1*s*, Pb 4*f*, and Br 3*d* before and after Al_2_O_3_ deposition (Fig. 2a–c). The intensity of the N 1*s* peak is significantly weakened due to CH_3_NH_2_ release. Furthermore, both Pb 4*f*_7/2_ and Pb 4*f*_5/2_ peaks (Fig. 2c) shift to lower binding energy values by 0.37 eV after the ALD process because of the Pb-rich surface defects bonded with adsorbed oxygen^39,40^, which can additively suppress nonradiative recombination on the perovskite surface. The surface microstructure of the MAPbBr_3_ SCTF is well retained after Al_2_O_3_ deposition, as revealed from the scanning electron microscopy (SEM) image (Supplementary Fig. 5b). Identical X-ray diffraction (XRD) patterns are observed in MAPbBr_3_ SCTF and MAPbBr_3_ SCTF/Al_2_O_3_ with the same growth orientation along the (h00) crystal plane, which not only reveals that the crystal phase of perovskite is well preserved during the ALD process, but also implies that the deposited Al_2_O_3_ is thin/amorphous (Supplementary Fig. 6).

A batch of MAPbBr_3_ SCTF/Al_2_O_3_ (0, 1, 5, and 10 nm) were fabricated by adjusting the ALD deposition cycles to unlock the effect of the Al_2_O_3_ layer on the PEC performance. Al_2_O_3_ thickness is achieved by controlling the number of ALD deposition cycles. Approximately 0.1 nm of aluminum oxide was deposited per cycle (for instance, 50 cycles, namely 5 nm). As shown in Fig. 2e, the saturated photoanode current density of the pristine MAPbBr_3_ SC film is ~1.2 mA cm^−2^ at 0.4 V vs. Ag/AgCl (0.53 V vs. SHE, Supplementary Fig. 7), which gradually increases with thickening Al_2_O_3_ and reaches a maximum value of 5.5 mA cm^−2^ at 5 nm of Al_2_O_3_. The enhanced PEC performance affirms that the ultrathin Al_2_O_3_ layer would not hinder photogenerated holes injection from perovskite to the electrolyte due to charge tunnelling. Comparing with pristine MAPbBr_3_ SC, coating a thin Al_2_O_3_ overlayer with a thickness of 1–10 nm resulted in slight overpotential increasement. This is because that deposited Al_2_O_3_ can isolate the photoelectrode from the electrolyte, therefore the photocorrosion phenomenon of MAPbBr_3_ SC being oxidized can be effectively suppressed. Meanwhile, with the increase of the thickness of Al_2_O_3_ overlayer, the passivation of effect of MAPbBr_3_ SC surface is more sufficient, so that the thicker 10 nm Al_2_O_3_ can reduce the overpotential comparing to the thinner Al_2_O_3_ (1 and 5 nm). The specific passivation mechanism will be discussed later. However, further thickening of the Al_2_O_3_ layer (e.g., 10 nm) attenuated the photoanodic current density, probably due to the aggrandized charge transport resistance as a result of its intrinsic insulating properties. The PEC performance of MAPbBr_3_ SCTF/Al_2_O_3_ (5 nm) is ~10 times higher than that of a previously reported BiVO_4_/WO_3_ photoanode with a maximum photocurrent of 0.55 mA cm^−2^ at 0.5 V vs. SHE)^28^. We have also investigated the influence of Al_2_O_3_ thickness on the stability. The stability of the electrode gradually improved with the increase of the thickness of Al_2_O_3_ from 0 to 10 nm (Supplementary Fig. 8), since the deposited Al_2_O_3_ can isolate photoelectrode from the electrolyte.

**Fig. 3:**
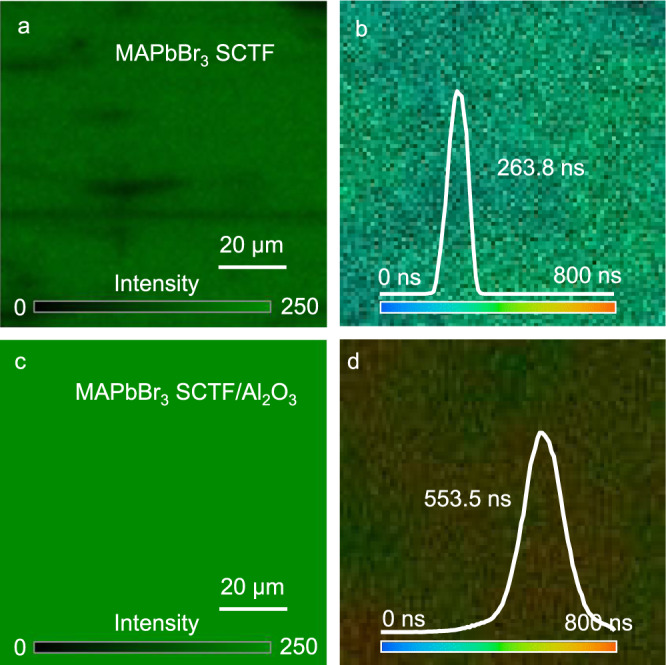
Fluorescence spectroscopic measurements. Confocal fluorescence intensity images (left) and fluorescence lifetime imaging microscopy (FLIM) images (right) of pristine MAPbBr_3_SCTF (**a**, **b**) and MAPbBr_3_SCTF/Al_2_O_3_ (**c**, **d**).

To identify the underlying reason for the increased PEC performance after coupling with an ultrathin Al_2_O_3_ layer, the optical properties of MAPbBr_3_ SCTF/Al_2_O_3_ were investigated. As shown in Fig. 2f, the PL emission intensity of the MAPbBr_3_ SCTF is significantly amplified after depositing 1-nm-thick Al_2_O_3_, and is further increased upon thickening of the Al_2_O_3_ layer to 5 and 10 nm. Time-resolved PL (TRPL) decay plots at the emission maximum (Fig. 2g) also demonstrate that all MAPbBr_3_ SCTF/Al_2_O_3_ samples decay sluggishly compared with pristine MAPbBr_3_ SCTF. Specifically, the fitted average PL decay time of MAPbBr_3_ SCTF/Al_2_O_3_ (5 nm) is twofold longer than that of pristine MAPbBr_3_ SCTF (Supplementary Table 1). This enhanced PL intensity and prolonged PL lifetime can be ascribed to the suppression of nonradiative recombination of electron-hole pairs in MAPbBr_3_ SCTF/Al_2_O_3_. In addition, spatial and temporal imaging PL emission was monitored with a sample size of 100 µm × 100 µm. Figure 3a, c show confocal fluorescence images of pristine MAPbBr_3_ SCTF and MAPbBr_3_ SCTF/Al_2_O_3_ (5 nm). Apparently, greater brightness is observed for MAPbBr_3_ SCTF/Al_2_O_3_, corresponding to stronger PL emission intensity. More importantly, brightness contrast appears with apparent darker spots in the images of pristine MAPbBr_3_ SCTF, but these spots are well dispelled after Al_2_O_3_ deposition, thus affording a uniform PL emission image (Fig. 3c). These darker spots on MAPbBr_3_ SCTF could be assigned to the region with higher surface trap-state density, and thus with active nonradiative charge carrier recombination^41^. Agreeing well with the TRPL test, fluorescence lifetime imaging microscopy (FLIM, Fig. 3b, d) data suggest a longer average PL lifetime for MAPbBr_3_ SCTF/Al_2_O_3_ (553.5 ns) than for pristine MAPbBr_3_ SCTF (263.8 ns). These results strongly affirm the passivation role of Al_2_O_3_, which effectively suppresses the trap states on the perovskite surface^42^.

**Fig. 4:**
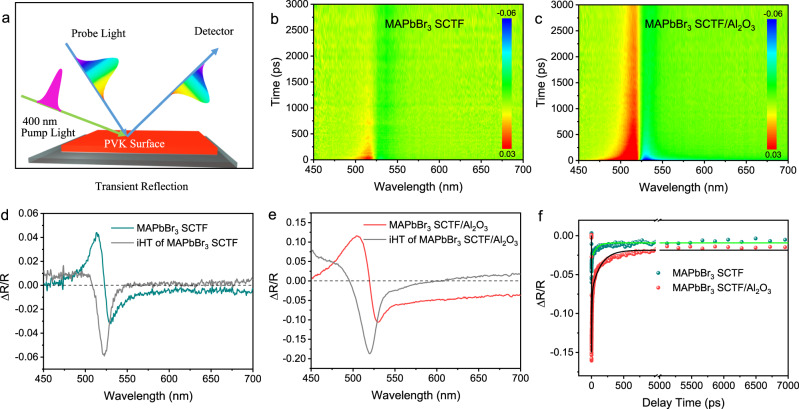
Transient reflection spectroscopic measurements. **a** Diagram of transient reflection (TR); TR spectra pseudocolour images, and TR spectra recorded at a delay time of 5 ps of pristine MAPbBr_3_ SCTF (**b**, **d**) and MAPbBr_3_ SCTF/Al_2_O_3_ (**c**, **e**). Kinetic fit of the data of these two SCTFs (**f**).

Different from the PL tests, transient reflection (TR) spectroscopy (Fig. 4a) can solely monitor the surface luminous and nonluminous species and provide detailed insight into the surface charge carrier dynamics, avoiding interferential signals from the bulk^37,43^. Both pristine MAPbBr_3_ SCTF and MAPbBr_3_ SCTF/Al_2_O_3_ feature a bandgap of 2.36 eV, corresponding to a UV–Vis absorption onset at 525 nm (Supplementary Fig. 9). Therefore, a 400 nm laser pulse with corresponding penetration depths of 80 nm was employed to excite only MAPbBr_3_^44^, and broadband continuum white light in the range of 450–700 nm was used as the probe pulse to monitor the reflection difference. Figure 4b, c show the pseudocolour TR spectra of pristine MAPbBr_3_ SCTF and MAPbBr_3_ SCTF/Al_2_O_3_, both characterized by an intense signal at approximately 520 nm. Accordingly, transient TR profiles recorded at a delay time of 5 ps also show a typical antisymmetric peak centred at 520 nm (Fig. 4d, e). To clearly reveal the transient absorption (TA) difference upon light excitation, the inverse Hilbert transform (iHT) was performed according to the Kramers–Kronig relationship^45^. As displayed in Fig. 4d, e, the resultant TA curves after iHT exhibit a ground state bleaching peak centred at ~520 nm, agreeing well with the absorption onset. Due to the restricted time window (≤7 ns) for the TR tests, the decay dynamics are mainly derived from the diffusion and recombination processes of surface photo-induced charge carriers^46^. The decay kinetics probed at 520 nm (Fig. 4f) were analysed and interpreted and show a very sluggish decay for MAPbBr_3_ SCTF/Al_2_O_3_ compared to that of the pristine MAPbBr_3_ SCTF (141 ps vs. 70 ps) due to the retardative surface trap-assisted recombination afforded by the ultrathin Al_2_O_3_ layer.

**Fig. 5:**
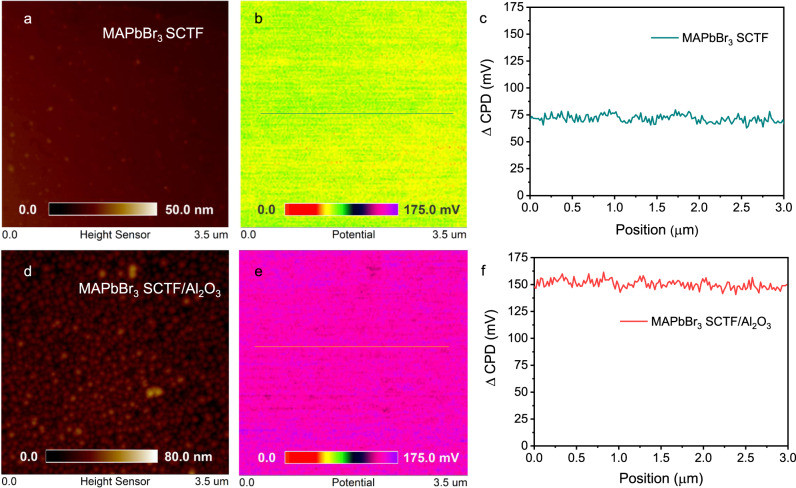
Kelvin probe force microscopy. AFM topography images, KPFM images and △CPD profiles (subtracting the potential in the dark from that under light irradiation) of pristine MAPbBr_3_ SC film (**a**–**c**) and MAPbBr_3_ SCTF/Al_2_O_3_ (**d**–**f**).

To further investigate the underlying effect of the Al_2_O_3_ layer on the electrical properties of the MAPbBr_3_ SCTF, Kelvin probe force microscopy (KPFM) was performed to probe the spatial charge transfer and separation on the perovskite. After subjection to the ALD 5 nm procedure, the MAPbBr_3_ SCTF surface appears slightly rougher with an increase in the root-mean-square value from 0.7 to 5.7 nm (Fig. 5a, d). Continuously distributed nanosized particles can be clearly observed in Fig. 5d, further corroborating that an ultrathin compact Al_2_O_3_ layer was uniformly deposited onto the MAPbBr_3_ SCTF, which is associated with island growth mechanism during ALD deposition of Al_2_O_3_^38^. The KPFM images and the corresponding line profiles of the surface photovoltage (SPV) difference are displayed in Fig. 5b, c, e, f. MAPbBr_3_ behaves as an *n*-type semiconductor according to UPS analysis, thus resulting in a space charge layer at the surface with an inherent electric field direction from the interior towards the surface^47^. The positive SPV values of MAPbBr_3_ SCTF and MAPbBr_3_ SCTF/Al_2_O_3_ indicated the photoinduced holes could transfer from the interior to surface of the sample. The SPV value of MAPbBr_3_ SCTF/Al_2_O_3_ is approximately 150 mV, twice higher than that of pristine MAPbBr_3_ PCTF (75 mV), indicating richer density of holes accumulated onto the surface, which is beneficial for enhancing the PEC performance.

### Investigation of the Ti layer effect on the PEC performance of MAPbBr_3_ SCTFs

**Fig. 6:**
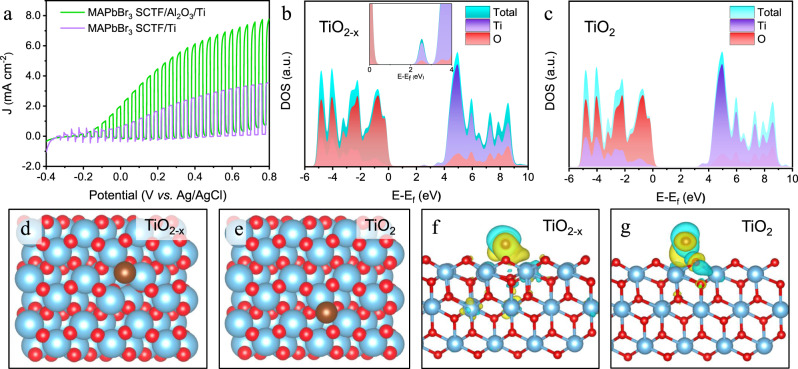
Ti layer effect on the PEC performance. **a** PEC behaviors of MAPbBr_3_ SCTF/Ti-based and MAPbBr_3_ SCTF/Al_2_O_3_/Ti-based photoelectrodes. The projected DOS of **b** TiO_2−x_ and **c** TiO_2_. DFT-calculated adsorption of Br on the surfaces of **d** TiO_2−x_ and **e** TiO_2_. **f**, **g** Corresponding electron density difference, where yellow indicates a gain of electrons and cyan indicates loss of electrons.

The above experimental results reveal that the Al_2_O_3_ passivation layer significantly enhances the PEC performance and the stability of the photoelectrode to some extent. However, due to its ultrathin thickness, long-term PEC stability is still challenging to maintain. Conductive overlayers have been widely proven to enhance the operating stability and even catalytic activity of semiconductor materials^8,13^. Thus, a 145-nm-thick Ti layer with a particle size of approximately 30 nm (Supplementary Fig. 10) was introduced onto MAPbBr_3_ SCTF/Al_2_O_3_ as a protective layer to avert direct interaction with the electrolyte. The MAPbBr_3_ SCTF/Al_2_O_3_/Ti shows almost the same XRD pattern as MAPbBr_3_ (Supplementary Fig. 11a). The reasons beyond the failure to observe the characteristic XRD pattern of Ti layer probably can be concluded as follows: (a) the high crystallinity of perovskite single crystal leads to its diffraction signals completely overwhelms the Ti signals; (b) amorphous nature of the deposited Ti layer. In order to find out the underlying reasons, we have tried to deposit Ti layer on the surface of quartz. The XRD results (Supplementary Fig. 11b) show that there are two new phases, which can be assigned to the Ti (PDF#01-074-7075) and rutile TiO_2_ (PDF#04-008-7856) with low crystallinity. The formation of TiO_2_ is ascribed to the oxidation of titanium by residual oxygen in the system during the evaporation process^48^. XPS spectrum (Supplementary Fig. 12) reveals that metallic Ti^0+^ (at 454.1 and 460.2 eV), Ti^3+^ (at 456.9 and 462.4 eV) and Ti^4+^ (at 458.7 and 464.4 eV) species are present, implying a TiO_x_-rich surface^49^. Figure 6a displays the current-potential curves of the MAPbBr_3_ SCTF/Al_2_O_3_/Ti-based PEC cell under chopped illumination. After incorporating the Ti layer on the MAPbBr_3_ SCTF/Al_2_O_3_ photoanode, the onset potential (~0.2 mA cm^−2^)^50^ was negatively shifted by 120 mV. The photocurrent was saturated for MAPbBr_3_/Al_2_O_3_ (5 nm) at 0.4 V vs. Ag/AgCl with 5.5 mA cm^−2^. In contrast, the photocurrent is not saturated for MAPbBr_3_ SCTF/Al_2_O_3_/Ti throughout all the test voltages from −0.4 to 0.8 V, reaching 7.8 mA cm^−2^ at 0.8 V vs. Ag/AgCl. To confirm the catalytic activity of Ti layer, the electrochemical behavior of FTO/Ti and FTO was also studied (Supplementary Fig. 13). Compared with the weak anodizing current of the FTO electrode, the FTO/Ti electrode exhibited remarkable electrocatalytic activity towards bromine oxidation with a near-zero overpotential and an onset potential of 0.73 V vs. SHE (0.60 V vs. Ag/AgCl). This result indicates that holes can easily be injected into the electrolyte and immediately participate in the chemical reaction due to the co-catalyst function of the Ti layer.

Density functional theory (DFT) calculations were conducted to shed light on the impact of Ti species on the electronic properties of the materials^51^. As shown in Supplementary Fig. 14, the calculated density of state (DOS) of Ti is continuous owing to its intrinsic metallic nature. Figure 6b, c illustrate that Ti^3+^ produces a higher density of oxygen vacancies and rearranges the band structure. The interaction between the Br atom and the three-atomic-layer p(3x) surface was probed (Fig. 6d, e). The calculated Br adsorption energy is more negative for TiO_2_ (−3.61 eV) than for TiO_2−x_ (−0.57 eV), implying that TiO_2−x_ has more potential as a Br oxidation catalyst. Upon adsorption, Br atoms suffer from charge redistribution and form chemical bonds with Ti atoms. The length of the Ti–Br bond on the TiO_2−x_ surface (2.59 Å) is estimated to be shorter than that on TiO_2_ (2.67 Å), agreeing well with its advantageous Br adsorption owing to the low-coordinated oxygen surface. The charge difference analysis in Fig. 6f, g displays a more significant charge transfer on the TiO_2−x_ surface than on the TiO_2_ surface. Based on Mulliken charge analysis, the Br atom is −0.26|e| on TiO_2−x_ but only −0.06|e| on TiO_2_. A similar trend has been observed based on Bader charge analysis, i.e., −0.54|e| on TiO_2−x_ and −0.26|e| on TiO_2_. The increased electron cloud density of Br favours the harvesting of holes during the subsequent oxidation process. Similar phenomena have also been observed in defect-rich TiO_2_, where the oxygen vacancies and Ti^3+^ ions facilitate more efficient charge transfer^52^ and serve as an intrinsic active species to increase the electrochemical^53^, photocatalytic, and PEC activities^54^. The current calculation results are consistent with the hypothesis that the introduction of catalytic active sites on the surface of the photoelectrode promotes the further improvement of PEC performance.

In addition to affording superior PEC performance, Ti overlayer can also protect the internal Al_2_O_3_ layer and perovskite layer. In order to further mitigate the influence of the electrolyte penetration from the edge, silicone sealant (Kafuter K-705) is used to encapsulate the edge of electrode. The resulted photoelectrode exhibited impressive stability for DMDF evolution, as displayed in Supplementary Fig. 15. The Br-mediated photogeneration of DMDF was carried out at 0.2 V vs. Ag/AgCl, which is lower than the potential required to initiate the electrochemical oxidation of bromine. The photocurrent density is well maintained even after 6 h of continual electrolysis at 0.2 V. The formation of DMDF is confirmed via mass spectrometry (Supplementary Fig. 16), and the Faraday efficiency is calculated to be as high as 93%, further promising its infinite potential as an effective and stable photoelectrode.

In summary, a MAPbBr_3_ SCTF-based photoanode could drive the conversion of furan to DMDF has been demonstrated. Combined investigations with FLIM and TR as well as KPFM characterizations demonstrated that an ultrathin Al_2_O_3_ layer effectively suppressed trap-assisted nonradiative charge recombination, resulting in a doubled surface charge carrier lifetime. Furthermore, the subsequent deposited Ti layer not only stabilized the MAPbBr_3_ SCTF by isolating perovskite from the electrolyte but also facilitated the bromine-mediated oxidation of furan owing to the Ti^3+^ catalytic active site. As a result, the highest photocurrent of 7.8 mA cm^−2^ at 0.8 V vs. Ag/AgCl was realized for the MAPbBr_3_ SCTF/Al_2_O_3_/Ti photoanode. The photoanode retained remarkable stability for more than 6 h under chopped light illumination. Considering the lead-related-toxic issues^55,56^, stable lead-free perovskite SCTFs should deserve more future research focus towards environment-friendly and efficient photocatalysts, whose application will be potentially expanded to currently still challenging biomass conversion and organic synthesis reactions in the near future.

## Methods

### Synthesis of methylammonium bromide (MABr)

A mixture of methylamine solution (30–33 wt% in methanol, Aladdin) and hydrobromic acid (48 wt% in water, Aladdin) with a molar ratio of 1:1.5 was stirred in an ice-water bath for 2 h. After that, MABr powder was precipitated by rotary evaporating the solvent at 50 °C and then purified by recrystallization. The collected MABr powder was dried in a vacuum oven for 24 h.

### Fabrication of MAPbBr_3_ single-crystal thin films (SCTFs)

MAPbBr_3_ SCTFs were grown onto TiO_2_-coated FTO glass substrates according to our previously reported method^31,32^. First, FTO glass substrates (7 Ω square^−1^, Nipponsheet Glass) were sequentially cleaned by ultrasonication in detergent water, alcohol, and acetone for 30 min per step. A compact TiO_2_ layer was subsequently deposited on the cleaned FTO glass via spin-coating of a TiO_2_ colloidal solution at 4 k rpm for 30 s, followed by an annealing process at 500 °C for 30 min. The annealed TiO_2_-coated FTO substrate was further immersed into a 0.04 M TiCl_4_ solution at 70 °C for 30 min before a secondary annealing process at 500 °C.

A space-limited model was assembled by sandwiching a thin polytetrafluoroethylene rectangular ring frame (20 μm in thickness) into two pieces of TiO_2_-coated FTO glasses. Then, the MAPbBr_3_ precursor solution, prepared by dissolving equimolar PbBr_2_ and MABr in DMF, was circulated into the limited space between the two substrates by a peristaltic pump (BT100-2J, Longer Pump). Note that the bottom FTO glass was drilled with two holes to facilitate the circulation of the perovskite precursor solution. The middle part of the model was locally heated to 95 °C for crystallization and then maintained at 80 °C for crystal growth. After ~72 h, the two FTO glasses were separated, with MAPbBr_3_ SCTFs grown in situ on the conductive substrate. The precipitated polycrystals were removed by γ-butyrolactone, and the residual solution was cleaned and removed by vacuum drying.

### Fabrication of MAPbBr_3_ PCTF

Equimolar MABr and PbBr_2_ were dissolved in DMF/DMSO (3:1, v–v) at a concentration of 1.2 M. An appropriate amount of perovskite precursor solution was spin-coated onto the substrate with a ramp rate of 3000 rpm, and 200 µL of chlorobenzene was dropped onto the spinning substrate 10 s after the spin-coating began. The obtained film was annealed at 120 °C for 10 min.

### Fabrication of MAPbBr_3_ SCTF/Al_2_O_3_/Ti

An ultrathin aluminum oxide layer was grown on the surface of MAPbBr_3_ SCTF by an atomic layer deposition (ALD) machine (ALD-SC6-PE, Syskey) with TMA and plasma oxygen as the element sources. In each ALD cycle, TMA was dosed into the chamber for 80 ms and purged out by Ar gas for 8 s, followed by dosing plasma O_2_ into the chamber for 3 s and purging out by Ar gas for 8 s. Approximately 0.1 nm of aluminum oxide was deposited per cycle. The ALD chamber was kept at a substrate temperature of 80 °C to achieve effective deposition of Al_2_O_3_. Another thin layer of Ti was further deposited on the aluminum oxide layer by the thermal evaporation deposition method (SKY Technology Development).

### Characterizations

XRD patterns were collected on a Rigaku Miniflex 600 X-ray diffractometer with Cu K_*α*_ radiation (*λ* = 1.5418 Å). X-ray photoelectron spectroscopy (XPS) was performed by means of a Thermo-VG Scientific ESCALAB 250 X-ray photoelectron spectrometer. The cross-sectional surface morphology and film thickness were examined by high-resolution field emission scanning electron microscopy (FE-SEM, Gemini 500). Atomic force microscopy (AFM) and scanning KPFM measurements were performed on a Bruker Dimension Fast Scan AFM system. UV–Vis absorption was obtained on an ultraviolet–visible spectrophotometer (Shimadzu UV-3600). Photoluminescence (PL) spectra were obtained on a fluorescence spectrophotometer (Edinburgh FLS980) with an excitation wavelength of 406 nm. Confocal fluorescence images and fluorescence-lifetime imaging microscopy (FLIM) images were recorded on a Zeiss LSM880 NLO microscope with an excitation wavelength at 405 nm. Transient reflectance spectra were measured on a Helios (Ultrafast Systems LLC) pump-probe setup in reflection mode. The pump beam at 400 nm was generated in a collinear optical parametric amplifier (OPerA Solo, Coherent) pumped by 800 nm fundamental pulses (100 fs, 1 kHz repetition rate, Astrella-Tunable-V-F-1k, Coherent). A sapphire crystal was used to generate a broad white light continuum probe spectrum. The TR signal changes were recorded in the visible region from 450 to 700 nm.

### DFT calculations

DFT calculations were carried out by means of the CP2K package^57^. The PBE functional^58^ with Grimme D3 correction^59^ and Goedecker-Teter-Hutter (GTH) pseudopotentials^60,61^ and DZVPMOLOPT-GTH basis sets^62^ were utilized to describe the system and the molecules, respectively. Unrestricted Kohn–Sham DFT was performed as the electronic structure method in the framework of the Gaussian and plane waves method^62,63^. A plane-wave energy cut-off of 500 Ry was employed. The adsorption energy (*E*_b_) is defined as:

1 $${{E}}_{\mathrm{b}} = E_{{\mathrm{Br/sur}}} - 1/2{{E}}_{{\mathrm{Br}}_2}(g) - {{E}}_{{\mathrm{sur}}},$$

where *E*_Br/sur_, ${{E}}_{{\mathrm{Br}}_2}$, and *E*_sur_ are the surface Br adsorption energy and the individual electron density of the molecule and surface, respectively. The charge density difference (Δ*ρ*) is defined as

2 $$\Delta \rho = \rho _{{\mathrm{Br/sur}}} - \rho _{{\mathrm{Br}}} - \rho _{{\mathrm{sur}}},$$

where *ρ*_Br/sur_, *ρ*_Br_, and *ρ*_sur_ are the electron density of surface-adsorbed Br and the individual electron density of Br and surface, respectively.

### Photoelectrochemical (PEC) measurements

PEC performance was measured on an electrochemical workstation (CHI660E, CH Instruments) at room temperature. A conventional three-electrode system with a Pt mesh as a counter electrode and Ag/AgCl_(sat KCl)_ as a reference electrode was adopted. To prevent dissolution and decomposition of perovskite, excessive MABr (2.02 g) was added to a mixed solution of anhydrous acetonitrile (45 mL) and anhydrous methanol (5 mL) as an anolyte. To prepare the target product of 2,5-dihydro-2,5-dimethoxyfuran, an additional 2.5 mL of furan (Innochem) was added. For the catholyte, 1-butyl-1-methylpyrrolidinium bis(trifluoromethylsulfonyl)imide (3.47 g, 99%, Shanghai Cheng Jie Chemical) and deionized water (0.5 mL) were added to anhydrous acetonitrile (50 mL). The linear sweep voltammogram (LSV) curves were measured at a scanning rate of 10 mV s^−1^ from −0.4 V to 0.8 V vs. Ag/AgCl under simulated solar illumination (Zolix, Gloria-X150A, 100 mW cm^−2^, AM 1.5G filtered, calibrated with a standard Si solar cell). The light on/off was regulated by the light chopping equipment (Perfect Light, PFS40A). After the photoelectrocatalysis test, 2 mL H_2_O was added into 1 mL of anode electrolyte and the solution was extracted with 0.5 mL ethyl acetate. The 2,5-dihydro-2,5-dimethoxyfuran in the top organic layer was quantitatively analyzed by gas chromatography using commercial 2,5-dihydro-2,5-dimethoxyfuran (TCI Shanghai) diluted with ethyl acetate as an external standard.

## Supplementary information

Supplementary Information

## Data Availability

The data that support the plots within this paper are available from the corresponding author upon request.
