# Supplementary material for: Surface passivated halide perovskite single-crystal for efficient photoelectrochemical synthesis of dimethoxydihydrofuran
Source: Nat Commun. 2021 Feb 22;12:1202. doi: 10.1038/s41467-021-21487-8 (PMC7900229; doi:10.1038/s41467-021-21487-8)
Supplement: Supplementary file 1 — Supplementary Information [file 41467_2021_21487_MOESM1_ESM.pdf]

Supplementary Information

Surface passivated halide perovskite single-crystal for efficient photoelectrochemical synthesis of dimethoxydihydrofuran

Xu-Dong Wang[1], Yu-Hua Huang[1], Jin-Feng Liao[1], Ze-Feng Wei[1], Wen-Guang Li[1], Yang-Fan Xu[1], Hong-Yan Chen[1] and Dai-Bin Kuang[1]*

[1]MOE Key Laboratory of Bioinorganic and Synthetic Chemistry, Lehn Institute of Functional Materials, School of Chemistry, Sun Yat-sen University, Guangzhou 510275, P. R. China.
*Corresponding author: kuangdb@mail.sysu.edu.cn.

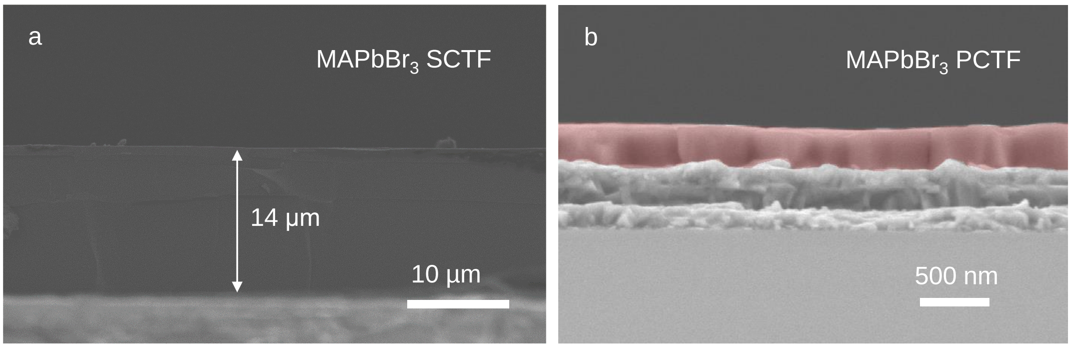

Supplementary Figure 1. Cross-sectional SEM images of $MAPbBr_3$ SCTF (a) and PCTF (b).

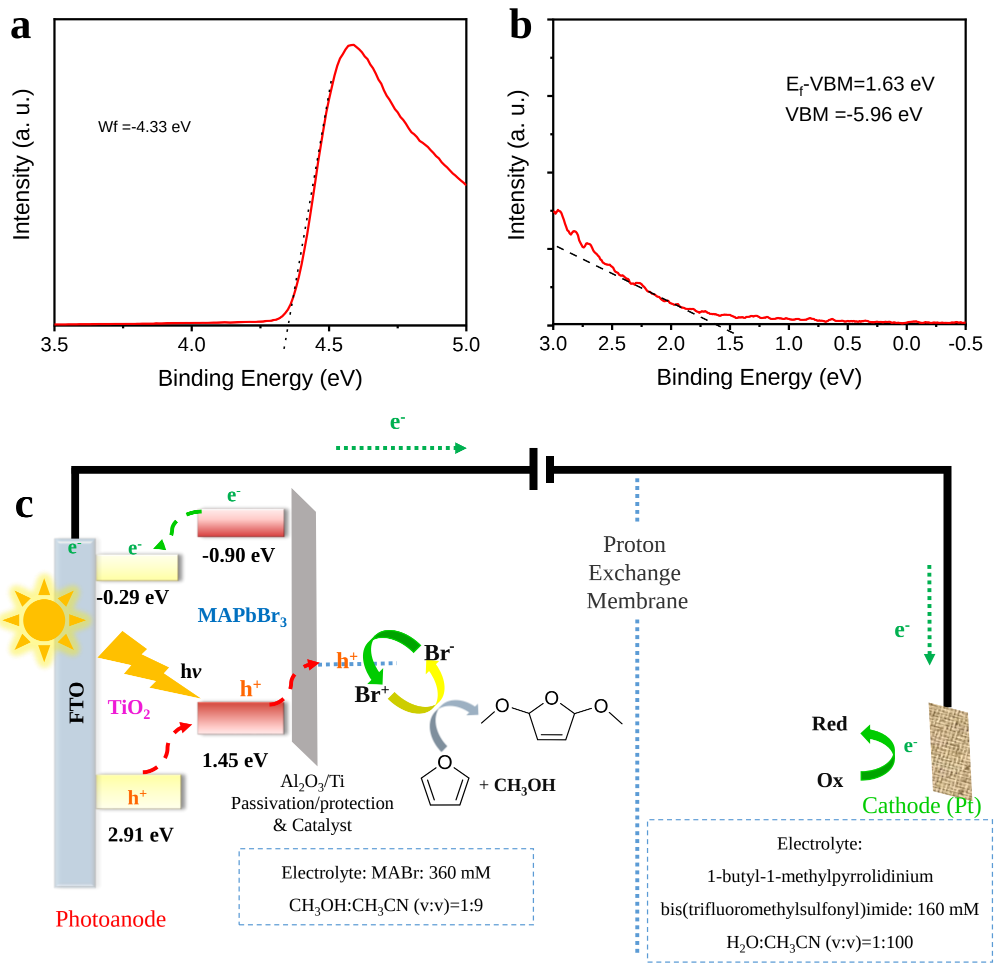

Supplementary Figure 2. (a, b) UPS spectra of $MAPbBr_3$ SCTF. (c) Diagram of the energy band structure and plausible mechanism for photoelectrochemical oxidation of furan with $MAPbBr_3$ SCTF as photoanode and Br^+/Br^- as a mediator.

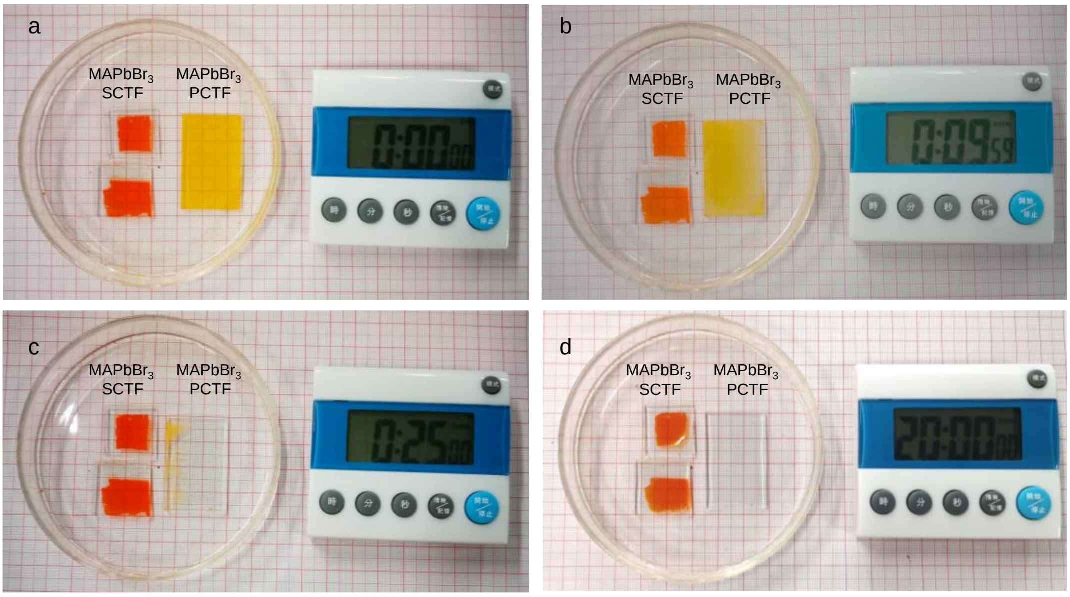

Supplementary Figure 3. Photographs of $MAPbBr_3$ SCTFs (14 μm) and PCTF (300 nm) after immersed in acetonitrile/methanol electrolyte for different period.

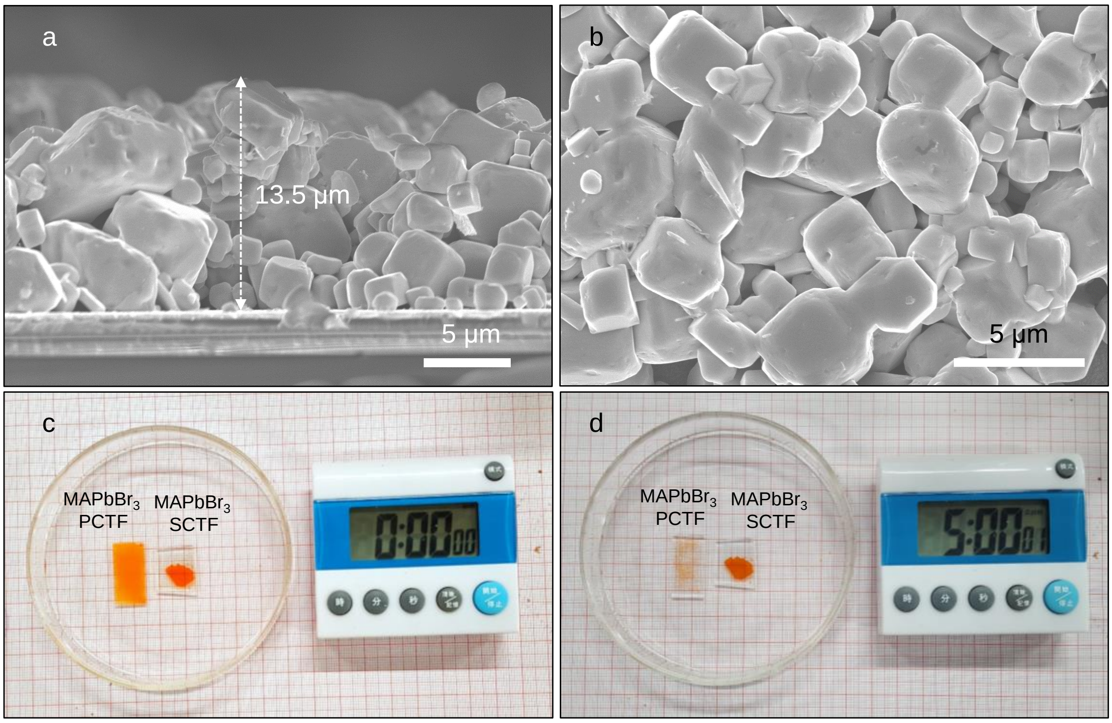

Supplementary Figure 4. Cross-sectional (a) and top view (b) SEM images of $MAPbBr_3$ PCTF with 13.5 μm in thickness. (c, d) Photographs of $MAPbBr_3$ PCTF (left) and SCTF (right) with similar thickness after immersed in acetonitrile/methanol electrolyte for 5h. $MAPbBr_3$ PCTF with 13.5 μm was fabricated by centrifugal deposition method. Firstly, 1 mL of $MAPbBr_3$ (1.5 M) DMF solution was added into 10 mL of toluene. After continuous ultra-sonication for 10 min, the as-prepared $MAPbBr_3$ suspension was cast onto an FTO substrate spinning at 9000 rpm for 3 min. The final $MAPbBr_3$ PCTF was dried under 70 °C for 30 min.

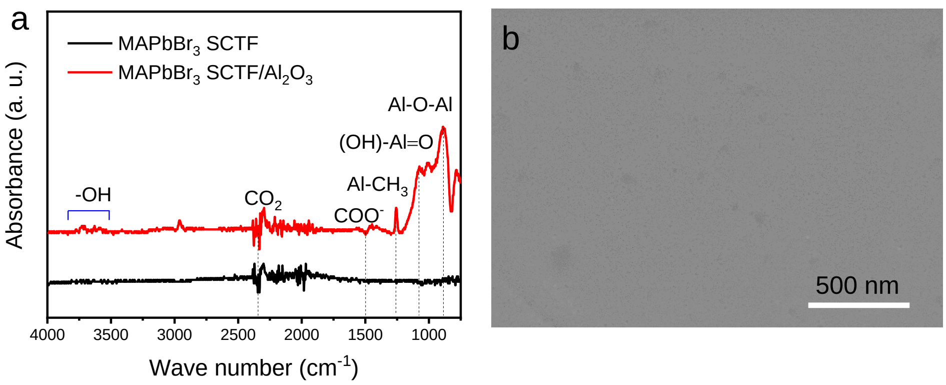

Supplementary Figure 5. Fourier transform infrared spectra of pristine $MAPbBr_3$ SC film and $MAPbBr_3$ SCTF /Al_2O_3 (a); SEM image of $MAPbBr_3$ SCTF/Al_2O_3 (b).

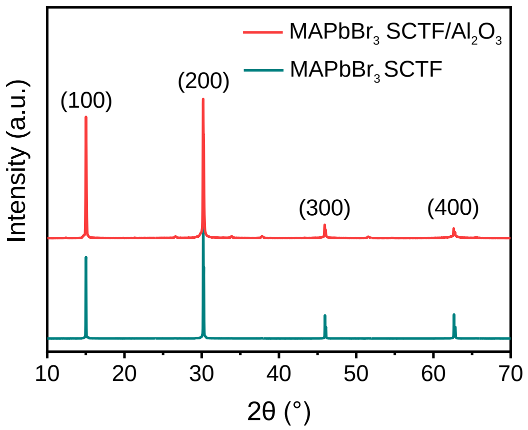

Supplementary Figure 6. XRD patterns $MAPbBr_3$ SCTF and $MAPbBr_3$ SCTF/Al_2O_3.

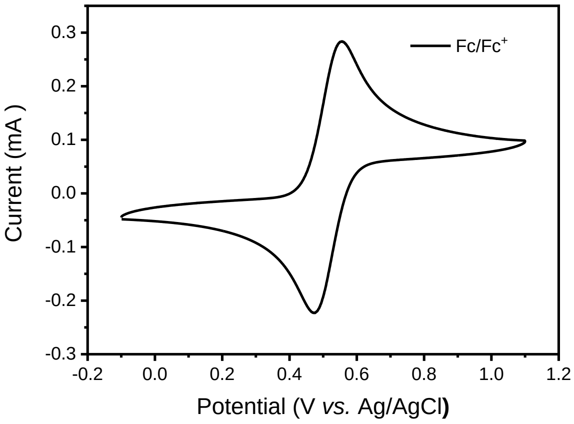

Supplementary Figure 7. Cyclic voltammograms for ferrocene/ferrocenium in 0.1 M $TPAPF_6$ acetonitrile solution at 50 mV s^{-1}. The reference electrode was calibrated towards the ferrocene/ferrocenium (Fc/Fc^+) redox couple (measured 0.51 V), $E_{SHE}= E_{Ag/AgCl}+ (0.64-0.51)V$.

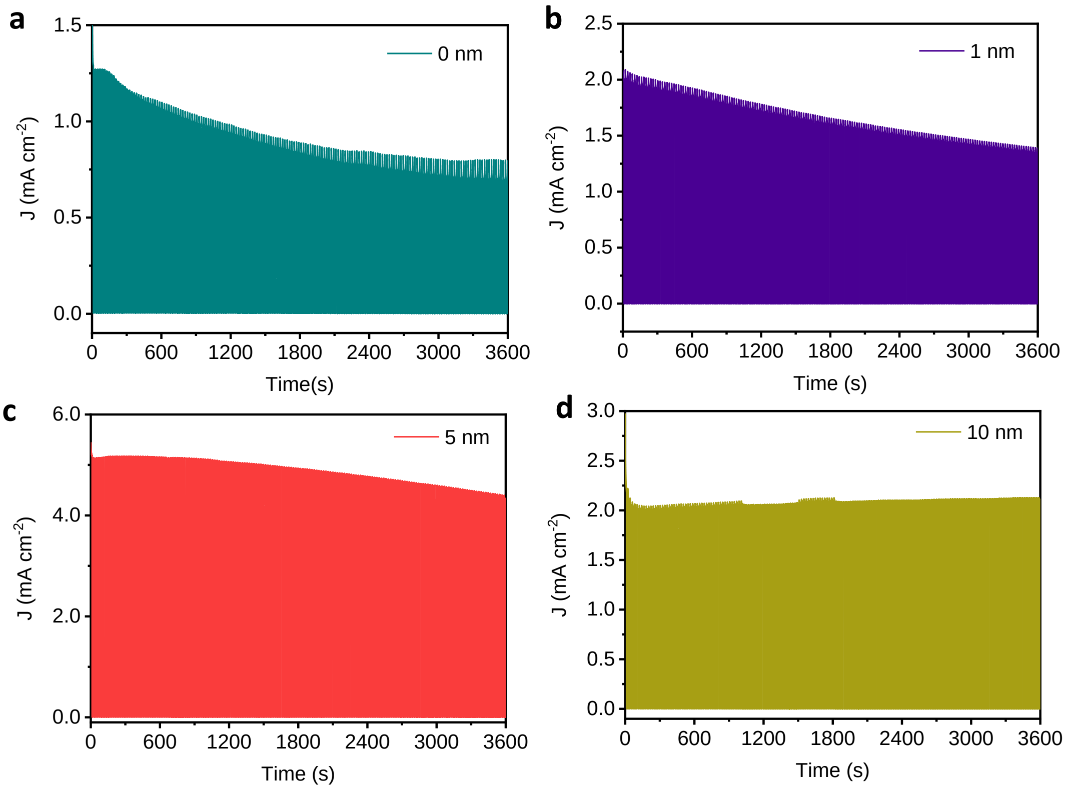

Supplementary Figure 8. Chronoamperometric traces of $MAPbBr_3$ SCTF/Al_2O_3-based photoelectrodes with different thicknesses of Al_2O_3, recorded at an applied potential of 0.4 V *vs*. Ag/AgCl.

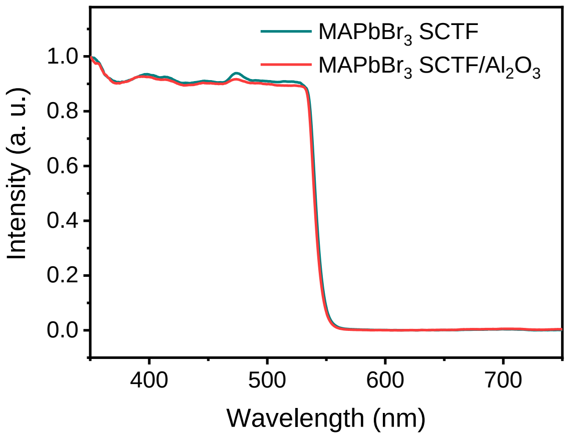

Supplementary Figure 9. UV-Vis absorption spectra of pristine $MAPbBr_3$ SCTF and $MAPbBr_3$ SCTF/Al_2O_3.

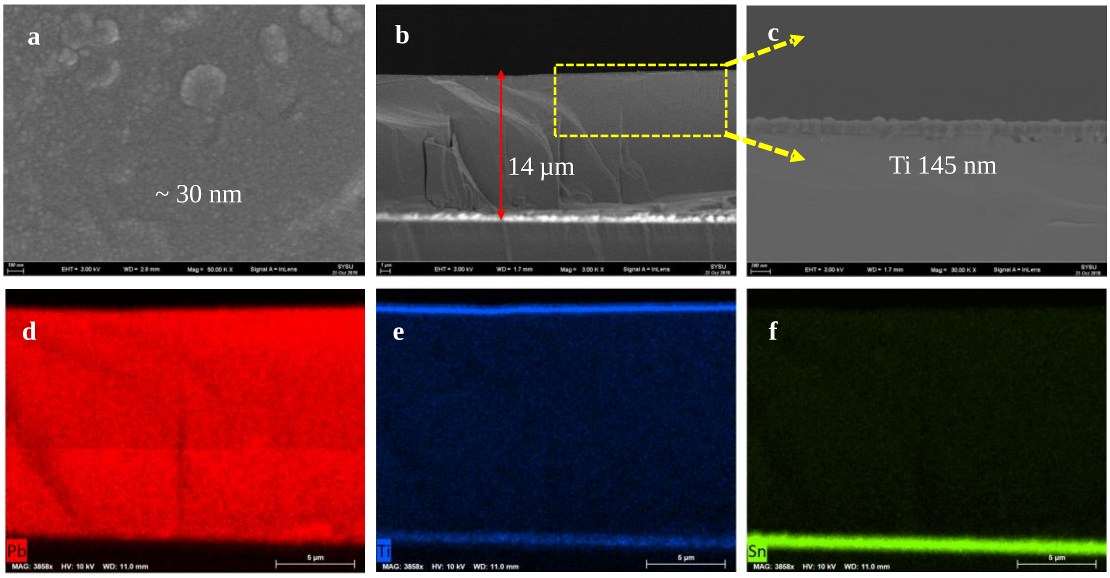

Supplementary Figure 10. Top- (a) and sectional-view (b, c) SEM images of FTO/TiO_2/$MAPbBr_3$ SCTF/Al_2O_3/Ti. Elements distribution in FTO/TiO_2/$MAPbBr_3$ SCTF/Al_2O_3/Ti film: Pb (d), Ti (e) and Sn (f).

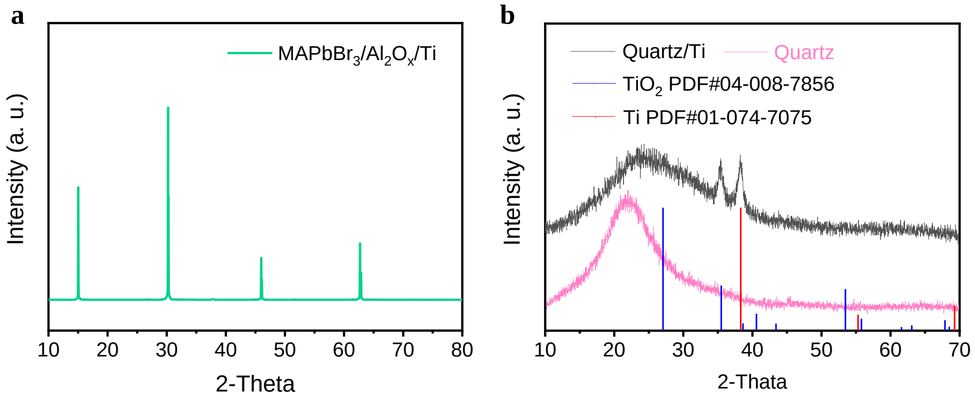

Supplementary Figure 11. XRD patterns of (a) FTO/$MAPbBr_3$ SCTF/Al_2O_3/Ti, (b) Quartz and Quartz/Ti.

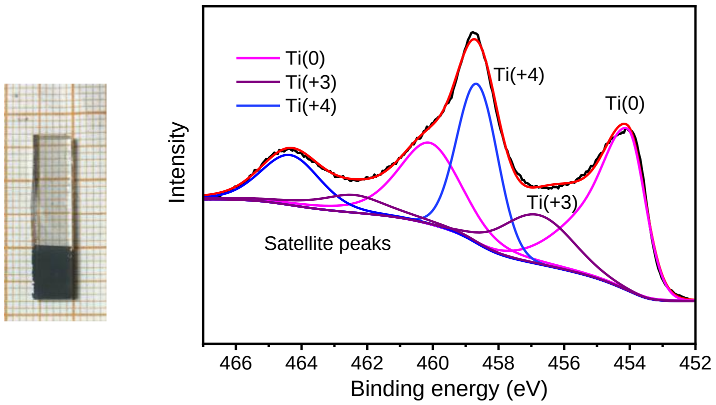

Supplementary Figure 12. Optical image (left) and surface XPS spectra of Ti peaks of glass/Ti film (right).

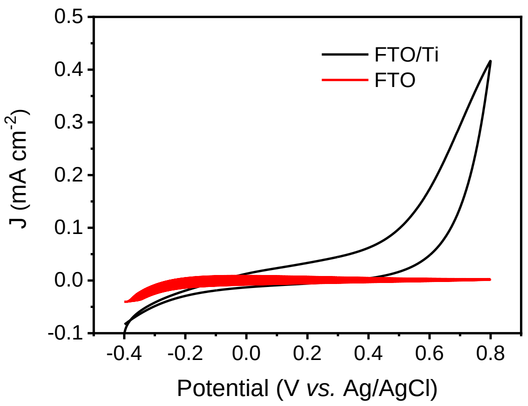

Supplementary Figure 13. Cyclic voltammograms for FTO and FTO/Ti electrodes at scan rate of 50 mV s^{-1}.

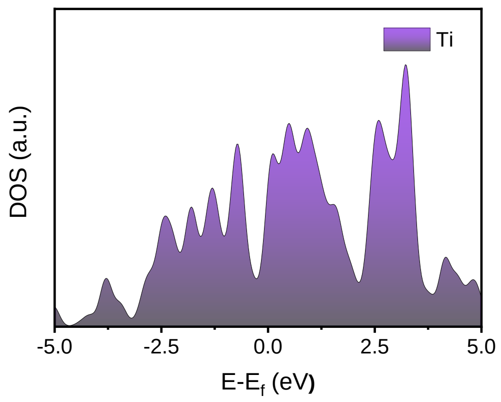

Supplementary Figure 14. The projected density of state of Ti.

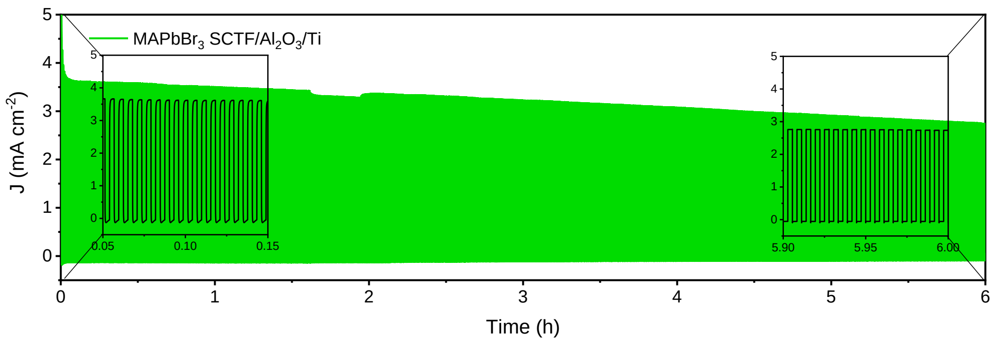

Supplementary Figure 15. $MAPbBr_3$ SCTF/Al_2O_3/Ti photoanode-based PEC cell under chopped illumination for 6 h with an applied bias of 0.2 V *vs*. Ag/AgCl.

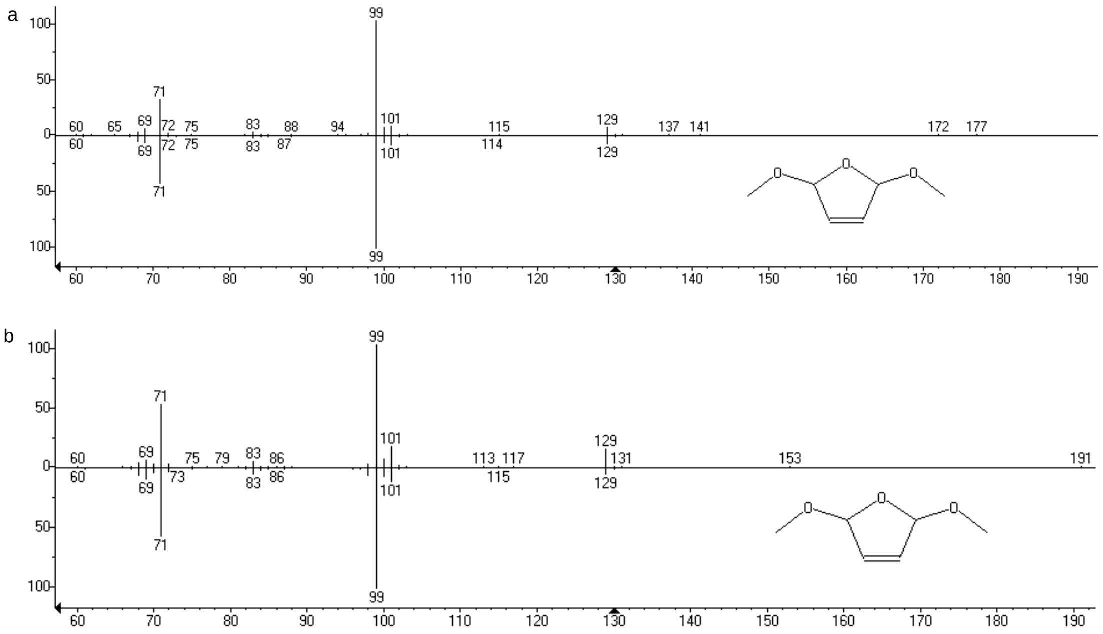

Supplementary Figure 16. The formation of DMDF isomerides comparing with the mass spectrometry spectral library (a, b).

Supplementary Table 1. Fitted lifetimes extracted from the TRPL spectra shown in Figure 2g.

$MAPbBr_3$/x-nm-thick Al_2O_3	**τ_1 (ns)**	**A_1 (%)**	**τ_2 (ns)**	**A_2 (%)**
0 nm	23.0	35.44	105.1	64.56
1 nm	57.4	37.49	189.3	62.51
5 nm	74.2	55.12	250.6	44.88
10 nm	74.5	85.01	284.9	14.99
